# Behavioral and Genetic Factors Associated with Successful Long-Term Cessation in Persons with HIV Who Smoke Cigarettes

**DOI:** 10.1155/2021/1894160

**Published:** 2021-12-10

**Authors:** Jonathan Shuter, H. Dean Hosgood, Ryung S. Kim, Kenny Ye, Cristina Montagna, Jidong Shan, Andrea H. Weinberger

**Affiliations:** ^1^Department of Medicine, Albert Einstein College of Medicine, Bronx, NY, USA; ^2^Department of Epidemiology and Population Health, Albert Einstein College of Medicine, Bronx, NY, USA; ^3^Department of Genetics, Albert Einstein College of Medicine, Bronx, NY, USA; ^4^Ferkauf Graduate School of Psychology, Yeshiva University, Bronx, NY, USA

## Abstract

**Background:**

Persons with HIV (PWH) smoke cigarettes at much higher rates than the general population in the US, and smoking is now the leading cause of death in US PWH. Efforts to control the tobacco use epidemic in PWH have met with limited success, and the factors associated with successful cessation are not well delineated. There is a particular dearth of knowledge regarding PWH ex-smokers who have successfully quit smoking cigarettes for the long term.

**Methods:**

We pooled data from three separate sources of PWH smokers and ex-smokers (reporting complete abstinence for ≥ one year with biochemical verification at the time of data collection) from New York City, collected sociodemographic and behavioral information from them in structured interviews, and obtained their DNA samples. Univariate and rigorous multivariate analytic strategies were employed to determine the sociobehavioral and genetic factors that distinguished PWH smokers from ex-smokers.

**Results:**

We compared 142 current/recent smokers to 52 biochemically confirmed ex-smokers. The mean age of the participants was 53.3 ± 9.9 years, 49.5% were female, and 76.3% were Black/African American. Successful quitters had significantly lower anxiety scores and were less likely to report hazardous alcohol use or to use marijuana or cocaine. On multivariate analysis utilizing a conservative analytic approach, of 156 single nucleotide variants (SNV) within 12 a priori candidate genes, only the 37148248 T->C variant of gene *SLC25A21* on chromosome 14 was associated with long-term cessation.

**Conclusions:**

In this study, we report behavioral variables associated with long-term abstinence in PWH ex-smokers, and we also report the first genetic correlation of successful cessation in a PWH population yet described.

## 1. Introduction

Approximately half of all persons living with HIV (PWH) in the United States (US) are active cigarette smokers [1, 2], and smoking has emerged as the leading cause of death in this group [3]. Numerous different tobacco treatment strategies have been employed in PWH with the goal of promoting cessation. Notwithstanding these efforts, an established approach to tobacco treatment for PWH remains elusive, and the largest meta-analysis on the topic performed to date concluded that some of the tobacco treatments increased short-term cessation, but none increased abstinence at the critical 6-month or later timepoints [4].

The factors that are associated with successful cessation, especially those with long-term cessation, in PWH smokers are not well-defined. Accurate profiles of those PWH smokers with a good chance of achieving cessation with tobacco treatment and of those with a poor chance could help the medical establishment better direct scarce resources. For example, a strategy of aggressive behavioral therapy combined with maximal pharmacotherapy may be most appropriate for individuals who have a high chance of success, whereas a greater emphasis on harm reduction (e.g., reducing daily cigarette intake or “cutting down”) and mitigation of other cardiovascular risk factors, such as hypertension, diabetes, hypercholesterolemia, and screening for lung cancer, may produce greater benefits for those who are unlikely to quit.

The role of genetics in delivering precision care to patients is steadily expanding and has become an important part of diagnostics, counseling, treatment, and clinical prediction across a broad range of medical disciplines [5]. The incorporation of pharmacogenomics into decision-making for patients is familiar to HIV care providers who have been screening abacavir candidates for the HLA-B∗5701 variant since 2007 [6].

Although genomics are not yet a standard element of tobacco treatment, the science of the genetics of tobacco use is rapidly advancing. Genetic variants associated with smoking and with quitting have been described [7], although not among PWH. It is possible that genetic predictors of cessation outcomes could be incorporated into models that estimate the likelihood of cessation. We are not aware of any prior research that has explored such associations in PWH smokers.

In the current study, we pooled several carefully characterized cohorts of PWH smokers and ex-smokers and sequenced a number of their genetic loci previously associated with tobacco use behaviors in other populations. Herein, we describe the behavioral and genetic correlates of successful long-term cessation in this very vulnerable group.

## 2. Materials and Methods

We assembled the patient sample for the current study from three separate sources.

Group 1: individuals who enrolled between May 2014 and August 2017 into a randomized controlled trial of Positively Smoke Free group therapy, our eight-session intensive behavioral tobacco treatment developed specifically for PWH smokers which was described in a prior publication [8]. This was a multicenter study that was conducted at three sites, two in the Bronx, New York, and one in Washington, DC. Study staff attempted to contact all enrollees at the two Bronx sites at least one year after their original study enrollment. Of the original 342 candidates, 11 had died since study enrollment, and 170 of the remaining 331 (51.3%) were successfully recalled for the current study.

Group 2: individuals who enrolled between December 2015 and December 2016 into a randomized controlled trial of Positively Smoke Free-Mobile, a cellphone-based tobacco treatment consisting of text messages, educational/motivational videos, and various on-screen tools to promote cessation in PWH smokers [9]. This was a single-center study conducted in the Bronx, New York. Study staff attempted to contact all enrollees at least one year after their original study enrollment. Of the original 100 candidates, 64 (64.0%) were successfully recalled for the current study.

Group 3: in order to increase the number of ex-smokers in the final sample, between November 2018 and March 2019, we recruited patients from Montefiore Medical Center's Center for Positive Living (CPL), a comprehensive HIV care facility, who reported a prior history of cigarette smoking (i.e., smoked >100 cigarettes in his/her lifetime) and had not smoked a cigarette in at least five years. Group 3 was comprised of a sample of 36 ex-smokers. Abstinence from smoking for all three groups was confirmed biochemically by exhaled carbon monoxide (ECO [coVita piCO+ Smokerlyzer®, coVita, Santa Barbara, CA] <10 parts per million [ppm]) prior to enrollment. New guidelines call for a lower cutoff (i.e., 5-6 ppm) for nonsmoker status, but these recommendations did not exist at the time that the present study was conducted [10].

The participants who were recruited in groups 1 + 2 were recalled to the CPL research suite to complete an audio computer-assisted self-interview (QDS-ACASI) to assess current smoking behaviors, undergo ECO testing, and submit a buccal swab for DNA collection.

The participants in group 3 completed an ACASI to assess their past smoking behaviors and other behavioral domains related to tobacco use, and they also underwent ECO measurement and buccal swab DNA collection.

Only participants whose DNA specimens were successfully sequenced were included in the final analyses. In the early stages of the study, when the methodologies for specimen collection, storage, transport, and DNA extraction were evolving, a number of samples provided inadequate DNA (41 in group 1, 32 in group 2, and 3 in group 3), and these participants were excluded.

We assembled a dataset for the entire sample that consisted of sociodemographic information, current (i.e., within the past 30 days) marijuana use, current (i.e., within the past 30 days) cocaine use, current hazardous alcohol use using the Alcohol Use Disorders Identification Test (AUDIT, with hazardous use defined as a score ≥ 8) [11], depression score using the Center for Epidemiologic Studies Depression Scale (CESD-20) [12], and anxiety score using the General Anxiety Disorder-7 (GAD-7) [13].

For the purpose of our analyses, current/recent smokers were defined as having smoked at least 100 lifetime cigarettes AND either admitting to having smoked a cigarette in the past 12 months OR having an ECO measurement ≥ 10 ppm. Ex-smokers all had ECO measurements < 10 ppm and denied smoking any cigarettes in the past 12 months among those in groups 1 and 2, and denied smoking any cigarettes in the past five years among those in group 3. ECO was measured at the time of the study visit. Repeated ECO measurements were not available to verify continuous long-term abstinence. The definition of ex-smoker in groups 1 and 2 was selected based on the observation made by Hughes et al. from the Society for Research on Nicotine and Tobacco's working group on abstinence measures that “epidemiologic studies often require 1-2 years of abstinence to avoid falsely declaring a temporary abstainer as a former smoker” [14]. According to standard terminology, after 12 months of abstinence, individuals are no longer considered “recent successful quitters” [15] and meet the definition of successful quitters. In group 3, for which we were not constrained by the end dates of the clinical trials, we elected to establish a more rigorous definition of ex-smoker, i.e., ≥five years of abstinence, since this duration of abstinence provided an even greater level of assurance that participants were long-term ex-smokers [16].

### 2.1. Genetic Data

We collected buccal swabs as a source of genomic DNA from all participants in order to explore associations between genetic variation and smoking status in PWH. We obtained the cells with Becton Dickinson CultureSwab™ Sterile Double Swab (Becton Dickinson, Franklin Lakes, NJ) swabbed across the buccal surfaces. Specimens were stored in a -70°C freezer. For DNA isolation, the tip of the collection swab was removed from the tube, dipped in 2 ml of Scope mouthwash and incubated overnight at 4°C after vortexing and the addition of 4 *μl* RNAse A, 300 *μl* of protease, and 2.5 ml of lysis buffer supplied with the QuickGene DNA whole blood kit (DB-L from Kurabo, Japan). Samples were next incubated at 56°C for 30 min before being processed for DNA extraction using a semiautomated nucleic acid isolation system (QuickGene 610 from Autogen). The quality of DNA was determined by the NanoDropTM 1000 Spectrophotometer (Thermo Fischer Scientific, Waltham, MA). The Qubit dsDNA HS Assay Kit (Life Technologies, Carlsbad, CA) was used to determine the concentration of each sample. We developed a custom next-generation sequencing (NGS) panel to map single-nucleotide variants (SNVs) in known exons and their promoter regions, of 12 candidate genes previously associated with tobacco use, addiction, and risk-taking behaviors (Supplemental Table I). We applied high-throughput sequencing to screen for mutations in *IREB2*, *SLC25A21*, *SEMA6D*, *CHRNA3*, *CHRNA4*, *CHRNA5*, *CHRNB4*, *PSMA4*, *DNMT3B*, *CADM2*, *CYP2A6*, and *HTR2B* using the Ion Proton Sequencer. Sequencing libraries were constructed starting from 10 ng of input DNA using amplification-based technology (Ion AmpliSeq Library Kit 2.0) as previously described [17]. Samples were multiplexed and sequenced in two P1 chips anticipating >500X sequencing depth for each sample. All samples were assigned a study ID, effectively blinding the samples of their smoking status through the DNA extraction and sequencing steps. Using our custom bioinformatics pipeline, we found a total of 759 single-nucleotide variants (SNVs) in the targeted regions.

For the clinical and behavioral data, categorical variables were analyzed using chi-square or Fisher exact test, where appropriate. Comparisons of means were accomplished using Student's *t*-test or Mann-Whitney *U* test, where appropriate.

For multivariate regression analysis of genetic and clinical data, we excluded 124 (out of 759) SNVs with less than 2% variation and further excluded 458 SNVs with ≥5% missing rate across samples because most of them were not in the targeted genomic regions. We performed complete case analysis with 157 out of 194 participants (37 participants who were missing data for any of the covariates considered in the regression model were excluded). After further excluding 21 SNVs which behaved identically with another SNV among these subjects, 156 SNVs entered the multivariate regression analysis. In order to analyze this dataset which was characterized by high levels of autocollinearity and a large number of predictor variables, logistic regression models with elastic net penalty were fit with four clinical covariates based upon univariate association with the outcome variable (current hazardous alcohol use, current marijuana use, current cocaine use, and anxiety score) and 156 SNVs (coded as 0, 1, 2 for homozygous absent, heterozygous, and homozygous present) of the 157 participants. Leave-one-out cross-validation was performed for each of 3 values of *α* (elastic net mixing parameter: 0, 0.5, or 1) and 100 values of *λ* (penalization parameter) to find the parameters that yielded the optimal area under the curve (AUC). The optimal parameters were *α* = 1 (which implies the least absolute shrinkage, or LASSO, model) and log(*λ*) = −2.974, which yielded an AUC = 0.663. *P* values were calculated according to recent developments in the statistical methods of postselection inference that account for variability in the process of covariate selection [18, 19]. We used Taylor's conservative approach to adjust the *P* values of the selected coefficients in the LASSO model [18, 20]. There are no established guidelines for estimating 95% confidence intervals around the adjusted odds ratios generated by this methodology, so none are presented. One clear strength of their method is that it controls the false discovery rate below the level of significance.

All aspects of the study were reviewed and approved by the Albert Einstein College of Medicine Institutional Review Board.

## 3. Results

The final study sample consisted of 194 participants (142 current/recent smokers and 52 ex-smokers), 129 in group 1 (111 current/recent smokers and 18 ex-smokers), 32 in group 2 (31 current/recent smokers and 1 ex-smoker), and 33 in group 3 (0 current/recent smokers and 33 ex-smokers). The mean age of the sample was 53.3 ± 9.9 years, 49.5% male, 49.5% female, 1% transgender, 76.3% Black/African American, 4.1% White, 19.6% other, undeclared, or of mixed race, 38.2% had not completed high school, 27.8% were high school graduates without posthigh school education, and 34% had partial or complete college education.

We initially performed analyses on the sociodemographic and behavioral variables separate from the genetic data.  Table 1 compares the characteristics of current/recent smokers (*N* = 142) with ex-smokers (*N* = 52) in our sample. Smokers who successfully quit (i.e., ex-smokers) were less likely to report current hazardous alcohol use, current marijuana use, and current cocaine use and had lower anxiety scores than current/recent smokers. In the multivariate logistic regression model, only current marijuana use (OR_adj_ 3.16 [95% CI: 1.33-7.52], *P* = 0.01) and current cocaine use (OR_adj_ 11.1 [95% CI: 1.44-83.3], *P* = 0.02) were significantly predictive of current/recent cigarette smoking.

In the multivariate analysis including genetic data, after adjusting for the other 156 distinct genetic variants and essential clinical variables, the C allele on chromosome 14 at position 37148248 was significantly associated, with an over 2-fold per-allele odds of being observed in nonsmokers (OR_adj_ = 2.02, *P* = 0.04) (Table 2).

## 4. Discussion

The effective management of the tobacco use epidemic in PWH has become a public health priority. Although there is a limited literature on characteristics associated with successful quitting in the short term [21–23], we know little about the sociodemographic, clinical, behavioral, and genotypic profiles of the subset of PWH who successfully quit smoking for the long-term. All PWH who smoke should be strongly encouraged to quit, and this encouragement should express itself in medical interventions to increase motivation to quit and to deliver appropriate behavioral therapy and pharmacotherapy [24]. However, tobacco treatment is not one-size-fits-all. Phenotypic characteristics, such as age group and pregnancy, inform the selection of treatment strategies. Nonmodifiable, genetically determined factors, particularly the nicotine metabolite ratio (the NMR), are known to affect response rates to various pharmacotherapies, and many experts support using NMR to guide pharmacotherapy selection [25]. Other genetic determinants also affect smoking and quitting behaviors [7]. The ability to stratify smokers into high and low likelihood groups for successful quitting would be valuable in optimizing the rational allocation of resources, e.g., by focusing intensive cessation treatments on those most likely to quit and by reducing harm, increasing motivation, and minimizing medical risks (with lung cancer screening, cardiovascular health assessments, etc.) in those who are unlikely to quit in the short term.

When we compared carefully characterized PWH successful long-term quitters to current PWH smokers, we found that the most influential predictors of tobacco abstinence within the range of sociodemographic and behavioral variables that we collected were current marijuana and current cocaine use. Cannabis and tobacco are frequently co-used, and their interrelationships are complex [26]. Active cigarette smokers are less likely to be successful in quitting their cannabis use [26]. Some studies have reported a lack of relationship between cannabis use and success at smoking cessation [27], and some have shown that cannabis users are less likely to quit smoking cigarettes than nonusers [28]. Interestingly, in a different study, we observed higher tobacco abstinence rates among active cannabis users in a sample of PWH who were followed prospectively in a trial of group tobacco cessation therapy [29]. Active cocaine use is a well-recognized impediment to tobacco cessation. In their analysis of the National Epidemiologic Survey of Alcohol and Related Conditions (total *N* = 43,093), Lopez-Quintero et al. found that cocaine dependence was among the most potent predictors of failure to overcome nicotine addiction in a national sample of US adults [30]. At least two prospective tobacco treatment trials in PWH similarly found that active cocaine use was a negative predictor of tobacco cessation in this group [8, 31].

The genetic findings that we report must be interpreted with caution given our small sample size. However, using statistical techniques that permitted adjustment for the large number of variants that we assayed, individuals having the thymidine to cytosine mutation at position 37148248 in the solute carrier family 25 member 21 (*SLC25A21*) gene on chromosome 14 had 2.02 times the odds of being successful long-term quitters with each additional copy of the C allele in a fully adjusted model. *SLC25A21* codes for a mitochondrial 2-oxodicarboxylate carrier whose role in tobacco use behaviors is undefined [32]. In the Tobacco and Genetics Consortium meta-analysis, based upon a sample of more than 74,000 individuals, the *SLC25A21* gene had the strongest statistical association with smoking cessation (*P* = 2.09 × 10^−08^) and was one of only two genes achieving genome-wide significance with the cessation outcome [33]. Notably, *SLC25A21* was the only one of these genes that was significantly associated with both quantity smoked (expressed in this study as either average or maximum number of cigarettes per day) and smoking cessation [33]. One earlier genome-wide association study also found a significant relationship between the *SLC25A21* gene and tobacco cessation [34]. Thus, notwithstanding the preliminary nature of our finding, there is substantial independent evidence lending credence to the possibility that genetic variation in *SLC25A21* may play a significant role in smoking and quitting among PWH. If verified in additional studies, variants of this gene could be utilized as predictors of quitting success or failure and possibly to identify molecular targets for future pharmacotherapeutics.

Our study had certain limitations that require mention. The sample size was small, and this limitation was worsened by the inadequate DNA collection that occurred early in the study in a sizable fraction of participants as we were refining our collection, storage, and transport techniques. Although we verified current abstinence biochemically at the time of the study visit, repeated ECO levels were not available to verify continuous abstinence in ex-smokers. Moreover, the ECO cutoff that we established for biochemical verification of nonsmoker status, although accepted at the time that the study was conducted, may have been too high according to new standards [10]. All of the participants were from the Bronx, which constrained the geographic and racial diversity of the sample. The research that we present in this paper should be viewed as preliminary, and it will hopefully prompt the future investigation of factors associated with successful long-term smoking cessation in larger, more diverse cohorts.

In the current study, we compared the sociodemographic and behavioral characteristics of a group of well-characterized PWH smokers to those of a group of PWH ex-smokers with biochemically confirmed, self-reported long-term abstinence. In addition to collecting their self-reported phenotypic data, we analyzed DNA specimens from the sample, focusing our assays on 12 genetic regions preselected for their a priori associations with cigarette smoking and other addictive behaviors. Consistent with earlier studies, we found that current marijuana use and current cocaine use were both associated with cigarette smoking. Ours is the first study to explore genetic associations with successful long-term cessation in PWH, and we describe herein a specific variant, 37146248 T→C of the *SLC25A21* gene on chromosome 14 that conferred greater than twice the odds of quitting per allele when adjusted for relevant behavioral variables as well as for the long list of other SNVs detected in our assays. It is notable that the *SLC25A21* gene is tightly associated with smoking cessation in large genome-wide association studies (GWAS) [33, 34]. This first foray into the genetics of cigarette smoking in PWH surely requires much additional research and independent validation in larger, more diverse samples, but our findings suggest that genetic markers may prove to be useful adjuncts in the understanding of prognostic categories of PWH smokers as we intensify our efforts to increase quit rates.

## Figures and Tables

**Table 1:** Characteristics of current/recent smokers compared with ex-smokers (*N* = 194).

Characteristic	Current/recent smokers (%) *N* = 142^a^	Ex-smokers (%) *N* = 52^a^	O.R. [95% C.I.]	*P*
Age (M. ± S.D.)	52.5 ± 10.0	55.2 ± 9.6	NA	0.11
Male gender	72/140 (51.4%)	24/52 (46.2%)	1.24 [0.65-2.33]	0.52
Race				
White	8 (6.8%)	0 (0.0%)		0.20
African American/Black	110 (93.2%)	38 (100.0%)	NA	
Latino/a	33/140 (23.6%)	12/52 (23.1%)	1.03 (0.48-2.18)	0.94
Education				
<High school graduate	57 (40.1%)	17 (32.7%)	NA	0.37
High school graduate	37 (26.1%)	17 (32.7%)		
Some college	35 (24.6%)	10 (19.2%)		
College graduate	13 (9.2%)	8 (15.4%)		
Hazardous alcohol use (AUDIT ≥ 8)	**25/131 (19.1%)**	**2/48 (4.2%)**	**5.43 [1.23-23.8]**	**0.02**
Current marijuana use^b^	**61/139 (43.9%)**	**9/52 (17.3%)**	**3.73 [1.69-8.26]**	**0.001**
Current cocaine use^b^	**35/139 (25.2%)**	**1/52 (1.9%)**	**17.2 [2.29-125]**	**<0.001**
Depression score	21.2 ± 12.2	19.2 ± 12.4	NA	0.33
Anxiety score	8.1 ± 6.3	5.3 ± 5.3	**NA**	**0.006**

Note: M.: mean; S.D.: standard deviation; O.R.: odds ratio; 95% C.I.: 95% confidence intervals; NA: not applicable; AUDIT: Alcohol Use Disorders Identification Test. ^a^Totals for each characteristic do not necessarily equate with the complete sample size because of missing data points. ^b^Current marijuana/cocaine use was defined as admitted use within the past 30 days.

**Table 2:** Multivariate logistic regression of factors associated with nonsmoking status (complete case *N* = 157).

Factor	OR_adj_^a^	*P*
Hazardous alcohol use (AUDIT ≥ 8)^b^	0.63	0.15
Current marijuana use^c^	0.55	0.51
Current cocaine use^c^	0.49	0.06
Anxiety score	0.99	0.80
*SLC25A21:* Chr.14 pos.37148248 T→C	2.02	0.04
*CADM2*: Chr.3 pos. 86118206 T→C	1.11	0.65
*CADM2*: Chr.3 pos. 86010739 T→A	1.36	0.19
*SLC25A21*: Chr.14 pos. 37641659 C→G	1.23	0.18
*CHRNA3*: Chr.15 pos. 78888349 A→G	0.70	0.06
*CHRNA3*: Chr.15 pos. 78909452 T→C	1.01	0.96
*DNMT3B*: Chr.20 pos. 31385158 C→G	0.85	0.63

Note: ^a^OR_adj_: adjusted odds ratio (for the genetic covariates the OR_adj_ is per each one allele increase); Chr.: chromosome; pos.: position; T: thymine; C: cytosine; A: adenine; G: guanine. ^b^AUDIT: Alcohol Use Disorders Identification Test; ^c^current marijuana/cocaine use was defined as admitted use within the past 30 days.

## Data Availability

Dataset is maintained internally at the Albert Einstein College of Medicine. Access to the dataset may be arranged through the corresponding author upon request.

## Supplementary Materials

Supplementary Materials Supplementary Table 1: genomic regions sequenced using the custom next-generation sequencing (NGS) panel.
