# Supplementary material for: Behavioral and Genetic Factors Associated with Successful Long-Term Cessation in Persons with HIV Who Smoke Cigarettes
Source: J Smok Cessat. 2021 Dec 10;2021:1894160. doi: 10.1155/2021/1894160 (PMC8683200; doi:10.1155/2021/1894160)
Supplement: Supplementary Materials — Supplementary Table 1: genomic regions sequenced using the custom next-generation sequencing (NGS) panel. [file 1894160.f1.docx]

**Supplemental Table 1**. Genomic regions sequenced using the custom next generation sequencing (NGS) panel.

| Gene | Chromosome | Chromosome Start | Chromosome End | Number of Amplicons | Total Bases | Number of Exons |
| --- | --- | --- | --- | --- | --- | --- |
| *SLC25A21* | chr14 | 37147120 | 37641870 | 30 | 4047 | 12 |
| *PSMA4* | chr15 | 78832741 | 78841568 | 13 | 1311 | 10 |
| *CHRNB4* | chr15 | 78916630 | 78933592 | 17 | 2508 | 6 |
| *CHRNA5* | chr15 | 78857856 | 78882983 | 20 | 3683 | 7 |
| *CHRNA3* | chr15 | 78885389 | 78913642 | 21 | 3635 | 9 |
| *SEMA6D* | chr15 | 47476397 | 48066425 | 52 | 7189 | 26 |
| *IREB2* | chr15 | 78730512 | 78793803 | 50 | 6587 | 22 |
| *CYP2A6* | chr19 | 41349437 | 41356357 | 20 | 1854 | 9 |
| *HTR2B* | chr2 | 231972944 | 231989829 | 15 | 2272 | 4 |
| *DNMT3B* | chr20 | 31350185 | 31397167 | 35 | 4870 | 24 |
| *CHRNA4* | chr20 | 61974656 | 61992753 | 33 | 5646 | 9 |
| *CADM2* | chr3 | 85008127 | 86123584 | 68 | 10011 | 14 |
